# Serial analyses of clinical spectra and outcomes in Chinese women with pregnancy-induced optic neuritis

**DOI:** 10.3389/fmed.2022.1067277

**Published:** 2022-11-25

**Authors:** Wenhao Bai, Mingming Sun, Honglu Song, Hongen Li, Xintong Xu, Xiyun Chen, Yixuan Zhao, Biyue Chen, Sheng Yao, Quangang Xu, Shihui Wei, Huanfen Zhou, Shengyuan Yu

**Affiliations:** ^1^Department of Neurology, The First Medical Centre of Chinese PLA General Hospital, Beijing, China; ^2^Senior Department of Ophthalmology, The Third Medical Centre of Chinese PLA General Hospital, Beijing, China; ^3^Department of Ophthalmology, Changping Maternal and Child Health Care Hospital, Beijing, China

**Keywords:** anti-aquaporin-4 antibody, myelin oligodendrocyte glycoprotein, optic neuritis, pregnancy, therapy

## Abstract

**Objective:**

This study aimed to investigate the clinical spectra and outcomes in pregnancy-related optic neuritis (ON).

**Methods:**

We analyzed the clinical subtype and prognosis of women with pregnancy-related ON in the neuro-ophthalmology department of the First Medical Center at the Chinese PLA General Hospital from January 2014 to December 2019.

**Results:**

A total of 54 patients, including 21 (38.9%) with idiopathic ON (ION), 27 (50.0%) with aquaporin-4 (AQP4)-ON, and 6 (11.6%) with myelin oligodendrocyte glycoprotein (MOG)-ON, who experienced 58 informative pregnancies and 67 episodes of pregnancy-related ON were assessed. Among the ON attacks, there were 11 (16.4%) during pregnancy and 56 (83.6%) within 1 year postpartum (PP1) or after abortion, including 33 (49.3%) in the first trimester. In total, 14 (25.9%) patients with ON onset before pregnancy had a higher relapse rate during PP1 than within 1 year before pregnancy (*p* = 0.021), and 24 (85.7%) eyes with ION and nine (100%) with MOG-ON had significantly better visual outcomes (p ≥ 0.5) than those with AQP4-ON (14, 35%) (*p* < 0.001 and *p* < 0.001, respectively). Two AQP4-ON patients had premature birth and low baby weight, respectively. There were no birth defects or stillbirths.

**Conclusion:**

The significantly increased relapse rate and numerous cases of ON after pregnancy suggest that delivery adversely affects the course of ON.

## Introduction

Optic neuritis (ON) is an acute inflammatory disease of the optic nerve that predominantly affects young adults, especially females of childbearing age. ON can occur independently or as an initial symptom of systemic demyelinating diseases, such as multiple sclerosis (MS) and neuromyelitis optica (NMO) (1). The aquaporin-4 antibody (AQP4-Ab) serves as an etiological factor of NMO and has gradually become a diagnostic and prognostic marker for ON in recent years (2, 3). A diagnosis of an NMO spectrum disorder (NMOSD) can be made in ON after one episode in the case of AQP4-Ab seropositivity, which indicates worse visual outcomes, a high recurrence rate, and the need for early immunosuppressive therapy as NMO differs from MS (4). The myelin oligodendrocyte glycoprotein antibody (MOG-Ab) as a biomarker for demyelination has revolutionized our understanding of ON (5, 6). There is no significant racial difference in MOG-ON patients, while AQP4-ON is more common in Asian and Latin American populations. The average age of onset of MOG-ON is younger, and AQP4-ON is more common in young and middle-aged women. The proportion of men and women in MOG-ON patients is similar, and the proportion of women in AQP4-ON patients is higher. When compared to MOG-ON, AQP4-ON associated myelitis myoclonus pain is more common (4–7). However, when compared to idiopathic ON (ION) and AQP4-ON, MOG-ON is strongly associated with bilateral ON and more severe optic nerve disk swelling, a relapsing disorder with better visual recovery that is frequently steroid-responsive and steroid-dependent (7).

Pregnancy induces a complex state of marked changes in the immunologic and hormonal milieu essential to the fetus (8). Thus, it is not surprising that pregnancy modulates the course of many autoimmune diseases (9). This issue has been addressed in various autoimmune diseases of the central nervous system, such as MS, NMO, and NMOSD, and it has been shown that disease onset is more likely to occur during the first year postpartum, especially during the first trimester, than during pregnancy (10–12).

To date, there have been few reports describing ON attacks, especially MOG-ON attacks during pregnancy (13). The detection of AQP4-Ab and MOG-Ab is crucial in patients with ON who are treated with different therapies and have different prognoses. Despite a higher relapse rate after the first episode, AQP4-ON had a higher relapse rate and worse visual recovery (4, 7). Rituximab has a good effect of preventing recurrence in AQP4-ON, but it is not sensitive in some patients with MOG-ON during a relapsing course when B- lymphocytes are controlled within the target value range. However, the prevalence of AQP4-Ab and MOG-Ab has never been assessed in pregnant women with ON. Therefore, we performed a cohort study to investigate the clinical spectra and outcomes of pregnancy-related ON attacks in Chinese women, which can provide novel insights into the management of ON in the peripartum period.

## Materials and methods

### Collection of patient records

The database of the department of neuro-ophthalmology in the First Medical Center at the Chinese People's Liberation Army General Hospital (PLAGH) was screened for patients with ON from January 2012 to December 2019. The diagnosis of ON was based on the criteria described in a previous study and included acute to subacute vision loss and the presence of at least two of the following: an afferent pupillary defect, color desaturation, pain upon eye movement, an abnormal visual evoked response, or a visual field defect without evidence of an infectious, metabolic, toxic, vascular, hereditary, or compressive etiology (14). MS, NMO, and NMOSDs were diagnosed according to the most recent international criteria (4, 15, 16).

The inclusion criteria for patients required an ON attack during pregnancy or within 1 year postpartum, while the exclusion criteria included a history of MS or NMO prior to a first episode of ON during pregnancy or within 1 year postpartum. All patients were followed for at least 2 years after delivery.

### Laboratory and neuro-ophthalmology examination

The patients underwent neuro-ophthalmic examination and laboratory tests, including cerebral spinal fluid (CSF) analysis. Patients' sera were tested for antinuclear antibodies (ANAs), lupus anticoagulant (A-dsDNA), extractable nuclear antigen antibodies (i.e., SSA and SSB), anti-cardiolipin antibodies (ACLs), beta-2 glycoprotein I antibodies (anti-b2GPI), anti-neutrophil cytoplasmic antibodies (ANCAs), rheumatoid factor (RF), and human leukocyte antigen-B27 (HLA-B27) in the PLAGH Examination Center for Biomedical Research. Serum AQP4-Ab and MOG-Ab examinations were performed using the best available method (i.e., a cell-based assay), as described previously by our group (17). The patients were classified into three groups, namely AQP4-ON, MOG-ON, and ION, based on their AQP4-Ab and MOG-Ab status.

The orbit and brain magnetic resonance (MR) images were made within 1 month of the ON attack in all patients, and spinal MR images were made in those patients with myelitis. Ophthalmological work-up included measurement of best corrected visual acuity, color desaturation test, test for afferent pupillary reaction (a pupillary lamp test), fundoscopy, visual field measurement, and optical coherence tomography for the detection of RNFL loss. Visual acuity was examined by the standard table of vision logarithms at 5 m. Patients unable to read any letters at 1 m were further examined by finger counts, hand movements, or light perception. Optical coherence tomography (OCT) examinations of retinal nerve fiber layer (RNFL) measurements were used to evaluate optic nerve atrophy. The follow-up data were obtained through return clinic visits by the patients and follow-up telephone surveys.

### Statistical analyses

All statistical analyses were performed using the SPSS 17.0 software (IBM Corporation, Armonk, NY). The Fisher exact test was used to compare categorical variables among groups, and an independent-samples *t*-test was used to compare the mean OCT measurements. Results with *p* < 0.05 were considered statistically significant.

## Results

By screening patient data from the PLAGH ophthalmology department, 242 female patients (including 172 pregnant patients) diagnosed with ON from March 2014 to May 2019 were identified. Among them, 54 patients experienced first or relapsing pregnancy-related ON attacks.

### Clinical spectra and ON attack distribution

Table 1 presents the clinical spectra and time distribution in patients with pregnancy-related ON. A total of 54 patients who experienced 58 informative pregnancies, including 27 (50.0%) ION, 21 (38.9%) AQP4-ON, and 6 (11.6%) MOG-ON patients, were assessed. The ages of the patients ranged from 22 to 37 years (mean 27.75 years). In total, 40 (74.1%) patients experienced their first ON attack during pregnancy or within 1 year after delivery or abortion, 14 (25.9%) patients experienced ON attacks before pregnancy, and three (21.4%) patients experienced ON attacks within 1 year prior to pregnancy.

**Table 1:** Clinical spectra and time distribution in patients with pregnancy-related ON.

	**Total**
Patients, *n*	54
Age at onset, y, mean ± SD (range)	27.93 ± 3.81 (22–37)
Age at delivery, y, mean± SD (range)	26.98 ± 4.50 (16–37)
Breastfeeding more than 1 month, n (%)	32 (59.2%)
AQP4-ON, n (%)	21 (38.9%)
MOG-ON, n (%)	6 (11.1%)
ION, n (%)	27 (50%)
ON attack before pregnancy, n (%)	14 (25.9%)
Within 1 year before pregnancy	3 (21.4%)
First ON attack at pregnancy or after delivery, n (%)	40 (74.1%)
Pregnancy times, n	58
Times of pregnancy related ON, n	67
In pregnancy, n (%)	11 (16.4%)
After abortion, n (%)	6 (9.0%)
After delivery, n (%)	50 (74.6%)

Among the 54 patients, there were 67 episodes of pregnancy-related ON. There were 11 (16.4%) ON attacks during pregnancy: 4 (6.0%) in the first trimester, 5 (7.4%) in the second trimester, and 2 (3.0%) in the third trimester.

**Figure 1:**
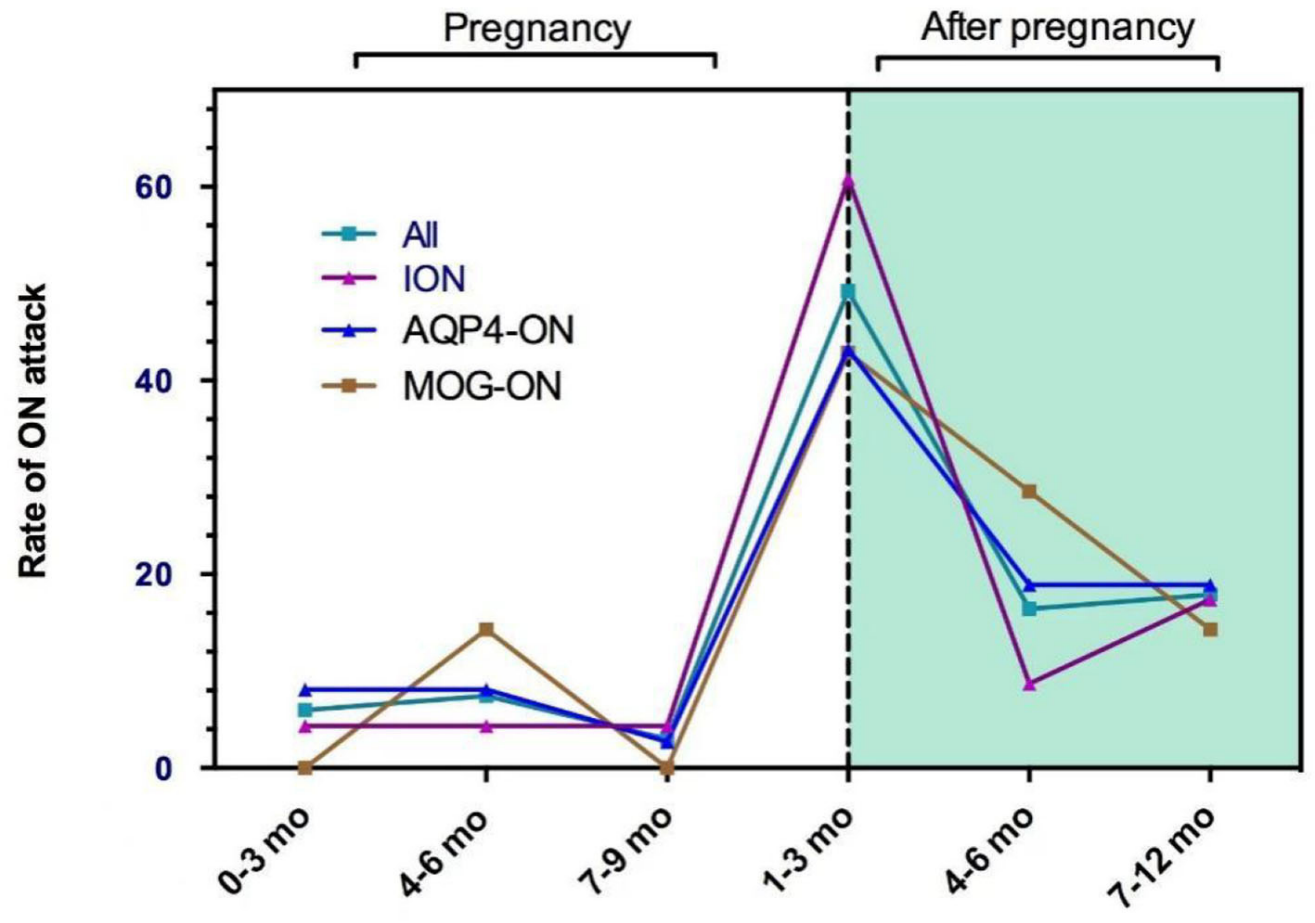
The pregnancy-related ON attacks in the ION, AQP4-ON, and MOG-ON groups.

There were 56 (83.6%) ON attacks within 1 year after delivery or abortion (50 ON attacks and 6 ON attacks, respectively), which included 33 (49.3%) in the first trimester, 11 (16.4%) in the second trimester, and 12 (17.9%) during 6–12 months after delivery or abortion. Figure 1 shows the pregnancy-related ON attacks in the ION, AQP4-ON, and MOG-ON groups. There were no significant differences among the three groups in different periods.

### Clinical profile and outcomes

Table 2 presents the clinical features and prognosis of pregnancy-related ON in the different groups. Of the pregnancy-induced ON attacks, 23 (42.6%) involved simultaneous or subsequent bilateral ON; 48 (88.9%) involved ocular pain; and 25 (46.3%) involved optic disk edema. MOG-ON had a higher rate of optic disk edema than ION and AQP4-ON (83.3 vs. 61.9%, *p* = 0.019 and 83.3 vs. 25.9%, *p* = 0.016, respectively). There were no significant differences in ocular pain among the three groups. A total of 9 (33.3%) patients with AQP4-ON had higher levels of abnormal autoimmune antibodies, including ANA, SSA or SSB, ACL, and β2-GPI than those with ION (0, *p* = 0.003). One patient with AQP4-ON had HLA-B27.

**Table 2:** The clinical features and prognosis of pregnancy-related ON in different groups.

**Clinical feature**	**All**	**ION**	**AQP4-ON**	**MOG-ON**	***P1 value***	***P2 value***	***P3 value***
Number of patients, n	54	21	27	6			
Bilateral attack, n (%)	23 (42.6%)	7 (33.3)	13 (48.1)	3 (50)	0.382	0.638	1.000
Ocular pain	48/54 (88.9)	18/21 (85.7)	24/27 (88.9)	6/6 (100.0)	1.000	1.000	1.000
Disk edema	25/54 (46.3)	13/21 (61.9)	7/27 (25.9)	5/6 (83.3)	**0.019**	0.628	**0.016**
Abnormal autoimmune antibodies, n (%)	10(18.5)	0	9 (33.3%)	1 (16.7%)	**0.003**	0.222	0.640
HLA-B27, n (%)	1 (1.9)	0	1 (3.7%)	0	/	/	/
VA at onset, eyes, n	77	28	40	9			
≤ 0.1	64 (83.1)	19 (67.9)	37 (92.5)	8 (88.9)	**0.021**	0.393	0.569
< 0.5–> 0.1	8 (10.4)	6 (21.4)	2 (5.0)	0	**0.057**	0.302	1.000
≥ 0.5	5 (6.5)	3 (10.7)	1 (2.5)	1 (11.1)	0.298	1.000	0.337
VA at recovery, eyes, n	77	28	40	9			
≤ 0.1	20 (26.0)	3 (10.7)	17 (42.5)	0	**0.006**	0.562	**0.019**
< 0.5–> 0.1	10 (13.0)	1 (3.6)	9 (22.5)	0	**0.039**	1.000	0.179
≥ 0.5	47 (61.0)	24 (85.7)	14 (35.0)	9 (100.0)	**0.000**	0.554	**0.000**
RNFL thickness, eyes, n	54	19	30	5			
Average thickness (mean ± SD)	70.20 ± 16.63	79.0 ± 18.69	62.20 ± 10.82	84.80 ± 11.37	**0.001**	0.518	**0.000**
Superior quadrant (mean ± SD)	81.07 ± 28.25	92.47 ± 34.02	71.27 ± 21.54	96.6 ± 19.09	**0.022**	0.799	**0.019**
Nasal quadrant (mean ± SD)	57.65 ± 10.31	61.53 ± 11.47	54.03 ± 6.49	64.6 ± 16.76	**0.050**	0.632	0.233
Inferior quadrant (mean ± SD)	87.22 ± 29.29	104.84 ± 29.77	72.13± 21.09	110.8 ± 16.63	**0.000**	0.674	**0.000**
Temporal quadrant (mean ± SD)	50.80 ± 15.16	55.16 ± 18.47	45.40 ± 7.69	66.6 ± 21.40	0.040	0.244	0.091
T2 lesions in orbit MRI	49 (90.7)	19 (90.5)	24 (88.9)	6 (100)	1.000	1.000	0.239

VA, visual acuity; RNFL, retinal nerve fiber layer.

Numbers in bold indicate p < 0.05.

Moreover, 37 (92.5%) eyes in the AQP4-ON group had severe vision loss (VA ≤ 0.1) compared to 19 (67.6%) eyes in the ION group (p = 0.011). In total, 24 (85.7%) eyes with ION and nine (100%) eyes with MOG-ON had significantly better visual outcomes (*p* ≥ 0.5) than those with AQP4-ON (14, 35%) (*p* < 0.001 and *p* < 0.001, respectively). There were still 17 (42.5%) eyes in the AQP4-ON group that experienced poor visual recovery (*p* ≤ 0.1), whereas only 3 (10.7%) eyes in the ION group and no eyes (0) in the MOG-ON group experienced similarly poor visual recovery (*p* = 0.006 and *p* = 0.019, respectively).

The average, superior, nasal, inferior, and temporal quadrants of RNFL thickness in the recovery stage (i.e., more than 3 months after the last ON attack) were significantly thinner in AQP4-ON than those in ION (*p* = 0.001, 0.022, 0.050, 0.000, and 0.040, respectively). The average, superior, and inferior quadrants of RNFL thickness in the recovery stage were significantly thinner in AQP4-ON than those in MOG-ON (*p* = 0.000, 0.019, and 0.000, respectively). Furthermore, no significant differences in the rate of optic nerve T2 lesions were presented between the three groups.

### The relapse rate in each period for 14 patients who became pregnant after the ON onset

**Figure 2:**
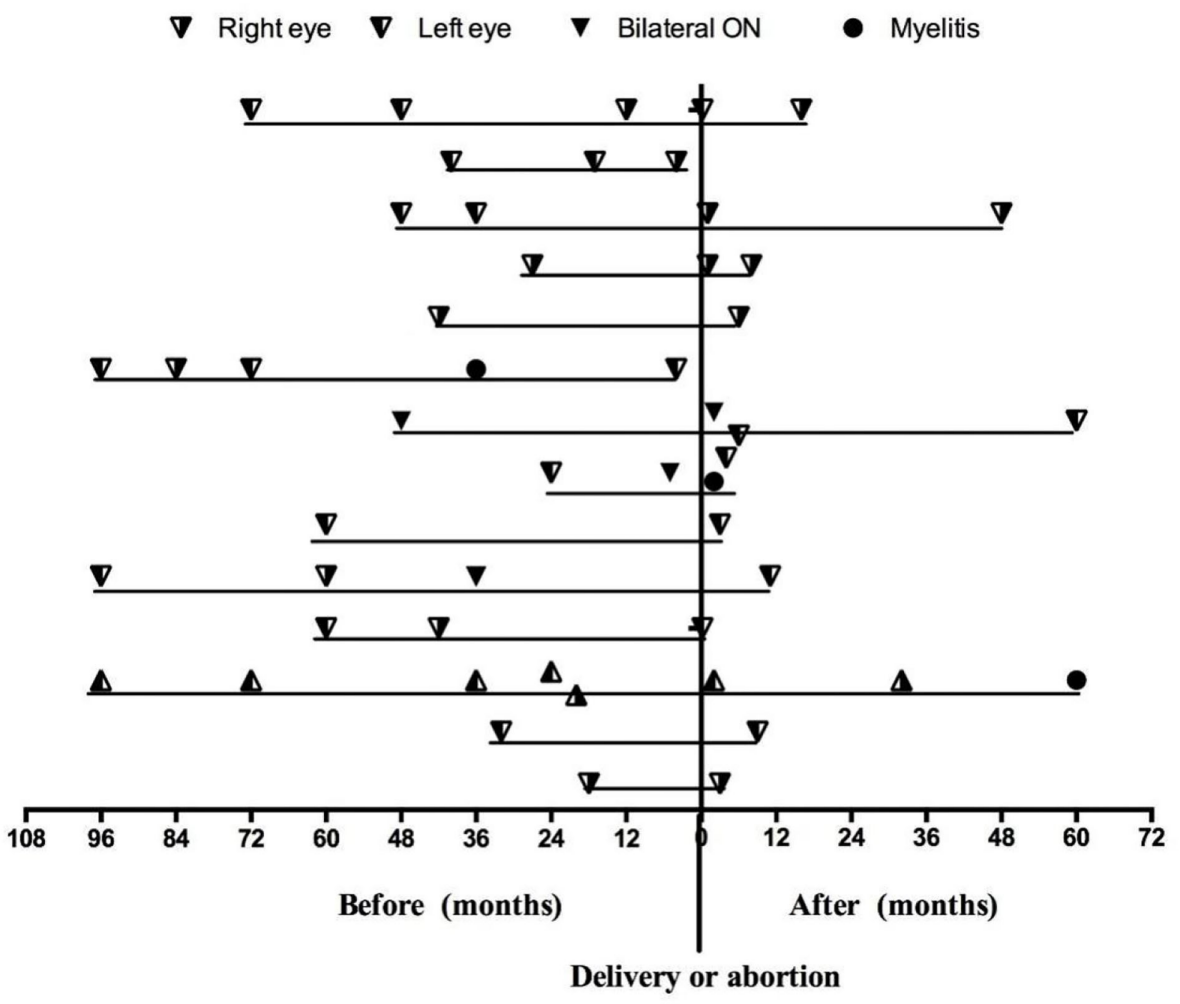
The relapses throughout the disease duration for 14 patients who became pregnant after ON onset.

**Figure 3:**
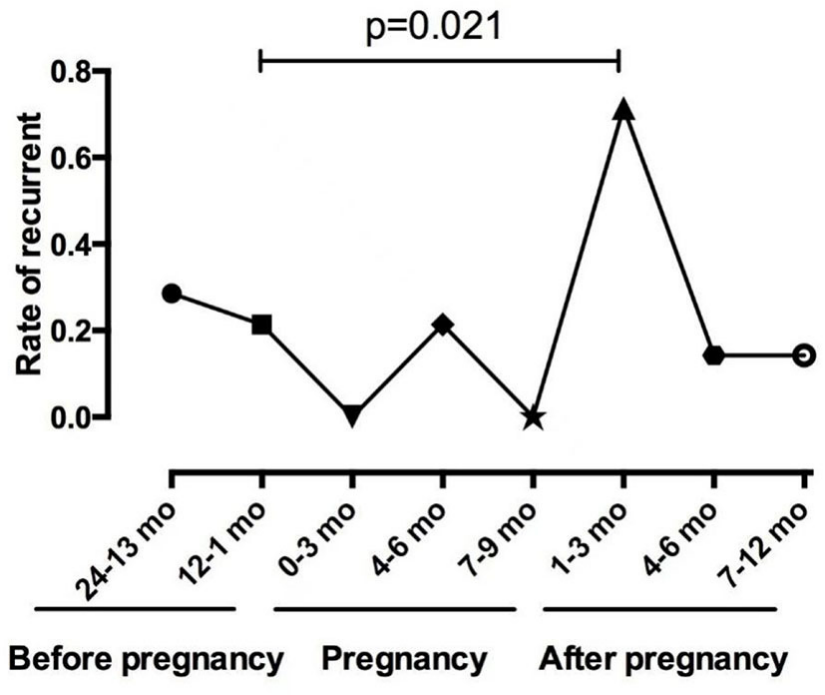
Comparison of the rate of recurrence in different periods of ON in the 14 patients.

Among the 14 patients who became pregnant after ON onset, 12 (85.7%) patients were AQP4-Ab seropositive, and two patients were AQP4-Ab and MOG-Ab seronegative. The mean duration of ON before pregnancy was 4.08 ± 2.39 years. Figure 2 shows the relapses throughout the disease duration for 14 patients who became pregnant after ON onset. Notably, three patients experienced an ON relapse during pregnancy, and 11 patients experienced an ON relapse after delivery, including two patients with two ON relapses and one patient with two ON relapses in two pregnancies. Figure 3 compares the rate of recurrence in different periods of ON in 14 patients. The results showed that the rate of recurrence was significantly higher during the first year postpartum than before pregnancy (*p* = 0.021).

### Treatment and prognosis

Table 3 presents the ON attacks and treatments during pregnancy. In total, eleven (16.4%) patients, including three ION patients, seven AQP4-ON patients, and one MOG-ON patient, experienced ON attacks during pregnancy. Three AQP4-Ab seropositive patients relapsed. Three patients (two AQP4-ON patients and one ION patient) did not receive treatment, and one ION patient experienced spontaneous visual recovery. One patient delivered a low birthweight infant, and one patient delivered premature infants, both of whom were AQP4-Ab seropositive. Notably, 43 patients who experienced ON attacks after delivery or abortion were all treated with IVMP, and they elected to discontinue breastfeeding during therapy. There were no birth defects or stillbirths. During more than 2 years of follow-up after delivery, six patients with AQP4-ON experienced transverse myelitis with a duration of 10–120 months.

**Table 3:** The treatment and delivery status of the patients with ON attack during pregnancy.

**Case**	**Serum**	**ON onset**	**First onset or relapse**	**Form of delivery**	**Immunosuppressive therapy during pregnancy**	**Neonates**
1	-	DP3	First	Nature birth	IVIg	Health
2	-	DP1	First	Induced abortion	IVMP after induced abortion	/
3	-	DP2	First	Nature birth	No treatment	Health
4	AQP4-Ab	DP2	Relapse	Nature birth	IVMP and IVIg	Health
5	AQP4-Ab	DP2	Relapse	Cesarean	Oral prednisone	Premature birth
6	AQP4-Ab	DP2	Relapse	Nature birth	IVMP	Health
7	AQP4-Ab	DP3	First	Nature birth	IVMP	Low baby weight
8	AQP4-Ab	DP1	First	Cesarean	No treatment	Health
9	AQP4-Ab	DP1	First	Drug abortion	IVMP after drug abortion	/
10	AQP4-Ab	DP1	First	Induced abortion	No treatment	/
11	MOG-Ab	DP2	First	Nature birth	IVMP	Health

IVMP, intravenous methylprednisolone; IVIg, intravenous immunoglobulin; DP1, the first trimester of pregnancy; DP2, the second trimester of pregnancy; DP3, the third trimester of pregnancy.

## Discussion

This cohort study examines the clinical spectra and outcomes of the largest group of pregnancy-related ON patients. A total of 54 patients, including 21 (38.9%) ION, 27 (50.0%) AQP4-ON, and 6 (11.6%) MOG-ON patients, who experienced 58 informative pregnancies and 67 episodes of pregnancy-related ON, were assessed, and the results indicated that AQP4-ON was the most common subtype in pregnancy-related ON. The detection of AQP4-Ab and MOG-Ab was crucial in patients with ON treated with different therapies (5, 18, 19). This pilot study illustrated the prevalence of AQP4-Ab and MOG-Ab in pregnant women with ON. By analyzing the clinical outcomes, we contributed novel insights into the management of ON in the peripartum period.

In this study, the patients had a higher rate of ON attacks within 1 year after delivery or abortion, especially in the first trimester (49.3%), and the results were similar among the different ON groups. The rate of ON recurrence was significantly higher in the first trimester after delivery than that during the year before pregnancy (*p* = 0.021) in the 14 patients who experienced ON attacks before pregnancy. The significantly increased relapse rate and numerous cases of ON onset after pregnancy suggested that delivery can adversely affect the ON prognosis. These viewpoints were consistent with the results reported for MS and NMOSDs (10, 11, 20, 21). In patients with MS, the annualized relapse rate (ARR) decreases significantly in the third trimester of pregnancy but increases in the first 3 months after delivery (22). In patients with NMO or NMOSDs, the relapse rate increases significantly during the third trimester and the first 3 months postpartum (23–25).

In this study, we illustrated the prognosis and outcomes of pregnancy-related ON. Forty-eight (88.9%) of the ON attacks involved ocular pain, which was more than that reported in previous reports. MOG-ON had a higher rate of optic disk edema than the ION and AQP4-ON groups, which corresponded to the results of our previous studies (7, 26). AQP4-ON had worse vision loss (VA ≤ 0.1) than ION (92.7 vs. 67.6%, p = 0.011) at the onset. MOG-ON and ION have excellent visual prognoses compared to AQP4-ON (100%, 85.7 vs. 35%). In addition, six patients with AQP4-ON experienced transverse myelitis during the follow-up period. The results above could indicate that pregnancy-related AQP4-ON had a more severe course and that the treatment of patients demands more attention.

Previous studies showed that women with seropositive AQP4-Ab NMO had a high rate of spontaneous abortion (27, 28). Animal models showed a high expression of AQP4 in the placenta, which suggests that NMO-IgG might induce placental necrosis, which can result in fetal death (29). Kim et al. analyzed 40 pregnancies in 26 women with NMOSD, which resulted in 14 abortions (one spontaneous and 11 elective) and 26 live births (1 with preterm birth and birth defects) (24). Fragoso et al. analyzed 17 Brazilian women with NMO during pregnancy or after delivery; 16 patients delivered healthy infants, and one patient had a miscarriage (28). In our study, one patient delivered an infant with low birth weight, and one patient gave premature birth, both of whom were AQP4-Ab seropositive. The patients with ON attacks during pregnancy accepted treatment with IVMP or IVIG (30), and none of the corresponding infants had birth defects or were stillbirths. However, due to the small sample size, these results might not represent actual fertility outcomes. Collectively, the potential complications should be considered when a woman with AQP4-Ab seropositive ON becomes pregnant. We can monitor the serum titers of AQP4 and serum IgG; if the AQP4 titer increases or the serum IgG decreases, it is recommended to choose intravenous immunoglobulin to reduce the recurrence rate.

This study had some major limitations. First, the single-center study design could lead to selection bias. Second, we were not able to evaluate the influence of BMI and breastfeeding on ON. Second, uni/multivariate logistic regression analyses have not been used to analyze the independent risk factors for pregnancy-related attacks, and AQP4-ab titer has not been monitored dynamically. Future studies should elucidate the roles of these factors, and how clinical practice could directly be improved through intensified screening and monitoring of pregnancy-related complications needs to be further elucidated in depth.

## Conclusion

This study summarizes the current knowledge on the interactions between ON and pregnancy. Our results might support the idea that pregnancy plays a pathogenic role in promoting pregnancy-related ON attacks and demonstrate that there is a significant increase in ON attacks after delivery, especially during the first trimester. AQP4-ON was the most common subtype of pregnancy-related ON, and MOG-ON had extremely better visual recovery. AQP4-ON should be given additional attention during the selection of treatment and pregnancy-related complications.

## Data availability statement

The raw data supporting the conclusions of this article will be made available by the authors, without undue reservation.

## Ethics statement

The studies involving human participants were reviewed and approved by the Chinese PLA General Hospital Ethics Committee. The patients/participants provided their written informed consent to participate in this study. Written informed consent was obtained from the individual(s) for the publication of any potentially identifiable images or data included in this article.

## Author contributions

Conceptualization and supervision: SYu, HZ, and SW. Funding acquisition: SYu and HZ. Writing—original draft and writing—review and editing: WB, MS, HZ, YZ, BC, and SYu. Project administration: SW and SYu. Methodology: WB, MS, and SYu. Formal analysis: WB and MS. Validation: HS. Visualization: YZ, BC, and HL. Software: XX and XC. Investigation: SYa, HS, HL, QX, WB, MS, XX, and XC. All authors contributed to the article and approved the submitted version.

## Funding

This study was supported by the Project of the National Natural Science Foundation of China (No: 82101110).

## Conflict of interest

The authors declare that the research was conducted in the absence of any commercial or financial relationships that could be construed as a potential conflict of interest.

## Publisher's note

All claims expressed in this article are solely those of the authors and do not necessarily represent those of their affiliated organizations, or those of the publisher, the editors and the reviewers. Any product that may be evaluated in this article, or claim that may be made by its manufacturer, is not guaranteed or endorsed by the publisher.
